# Genome-wide analysis of long noncoding RNA profiling in PRRSV-infected PAM cells by RNA sequencing

**DOI:** 10.1038/s41598-017-05279-z

**Published:** 2017-07-10

**Authors:** Jing Zhang, Pu Sun, Lipeng Gan, Weijie Bai, Zhijia Wang, Dong Li, Yimei Cao, Yuanfang Fu, Pinghua Li, Xingwen Bai, Xueqing Ma, Huifang Bao, Yingli Chen, Zaixin Liu, Zengjun Lu

**Affiliations:** State Key Laboratory of Veterinary Etiological Biology, Lanzhou Veterinary Research Institute, Chinese Academy of Agricultural Sciences, Lanzhou, 730046 China

## Abstract

Porcine reproductive and respiratory syndrome (PRRS) is a major threat to the global swine industry and causes tremendous economic losses. Its causative agent, porcine reproductive and respiratory syndrome virus (PRRSV), primarily infects immune cells, such as porcine alveolar macrophages and dendritic cells. PRRSV infection results in immune suppression, antibody-dependent enhancement, and persistent infection. Highly pathogenic strains in China cause high fever and severe inflammatory responses in the lungs. However, the pathogenesis of PRRSV is still not fully understood. In this study, we analysed the long noncoding RNA (lncRNA) and mRNA expression profiles of the HP-PRRSV GSWW15 and the North American strain FL-12 in infected porcine alveolar macrophages (PAMs) at 12 and 24 hours post-infection. We predicted 12,867 novel lncRNAs, 299 of which were differentially expressed after viral infection. The Gene Ontology (GO) and Kyoto Encyclopaedia of Genes and Genomes (KEGG) analyses of the genes adjacent to lncRNAs showed that they were enriched in pathways related to viral infection and immune response, indicating that lncRNAs might play regulatory roles in virus-host interactions. Our study provided information about lncRNAs in the porcine immune system and offers new insights into the pathogenic mechanism of PRRSV infection and novel antiviral therapy development.

## Introduction

Porcine reproductive and respiratory syndrome (PRRS) is the most economically significant infectious disease in the swine industry worldwide. It is characterized by respiratory tract illness in piglets and reproductive failure in sows. The etiologic agent, porcine reproductive and respiratory syndrome virus (PRRSV), is an enveloped, single-stranded positive-sense RNA virus that belongs to the family Arteriviridae^1^. Highly pathogenic porcine reproductive and respiratory syndrome (HP-PRRS) emerged in China and is associated with high fever, high morbidity, and high mortality^2^. Highly pathogenic PRRSV (HP-PRRSV) contains a discontinuous 30-amino-acid deletion in non-structural protein 2 (Nsp2) and has predominated in the field since the first outbreak^3^. PRRSV exhibits strict cell tropism; the main target cells are porcine alveolar macrophages (PAMs). PRRSV replicates in infected PAMs and causes changes in the morphology and function of PAM cells^4^.

Long noncoding RNAs (lncRNAs) are defined as non-protein-coding transcripts longer than 200 nucleotides. The majority of lncRNAs are 7-methylguanosine-capped at the 5′-end; some of them are also polyadenylated at the 3′-end^5^. Emerging evidence suggests that lncRNAs play regulatory roles in numerous physiological processes, such as gene imprinting, cell–cycle control, and embryonic development. Moreover, dysregulated expression of lncRNAs has been linked to human diseases including cancer and neurological disorders^6, 7^. Recently, numerous reports have demonstrated that lncRNAs are induced to modulate the innate and adaptive immune responses. In the innate immune response, lncRNA NKILA (NF-κB-interacting lncRNA) regulates transcription of NF-κB signalling components^8^. LncRNA NRAV (negative regulator of antiviral response) suppresses antiviral responses through down-regulation of interferon-stimulated gene transcription and promotes influenza A virus (IAV) replication and virulence^9^. LncRNA Nest expression increased susceptibility to Theiler’s virus in mice by altering the histone modification state at the IFN-γ locus^10^. LncRNA NEAT1 facilitates IL-8 production in response to influenza virus and herpes simplex virus infection^11^.

PRRSV strongly suppresses host innate and adaptive immune responses and results in persistent infection. Production of type I IFN is inhibited by Nsp1, Nsp2, Nsp4 and Nsp11 of PRRSV^12–16^. Despite suppression of key cytokines in the innate immune response, PRRSV also induces secretion of immunosuppressive cytokines such as IL-10 and TGF-β^1, 17–19^. In adaptive immunity, the earliest and strongest antibodies developed by infected pigs are against the N protein, but these initial antibodies do not exhibit protective effects and can be detrimental by mediating antibody-dependent enhancement^20^. In contrast, the neutralizing antibody response is weak and delayed^21^. Commercially available vaccines against PRRSV have been developed. However, they failed to provide complete protection, especially against genetically distinctive strains^22^.

Numerous tools are utilized to identify porcine genes that respond to infectious agents, such as proteomics, single-nucleotide polymorphism chips, and genome-wide association studies (GWAS)^23–25^. Studies have explored differentially expressed host mRNAs after PRRSV infection in pig lungs or PAM cells by transcriptome analyses (RNA-seq)^26, 27^. However, lncRNAs in the pig immune system have rarely been studied. In this research, we analysed the mRNA and lncRNA expression profiles of PAM cells infected with the different virulent PRRSV strains GSWW15 (GSWW) and FL-12^28, 29^ at 12 and 24 hours after infection using next-generation sequencing. The genomic features of lncRNAs and mRNAs were analysed, and in agreement with previous reports, the lncRNAs identified in our study were shorter, had fewer exons and were less conserved than mRNAs. We identified 299 lncRNAs that were differentially expressed after virus infection and annotated the possible functions of the predicted lncRNAs. Some were located in neighbouring genes involved in the virus infection pathway, indicating that lncRNAs might modulate the immune response against PRRSV by regulating up- or downstream genes. The roles of lncRNAs in the porcine immune system, which regulate host-viral interactions should be further studied. This research analysed long noncoding transcripts in PAM cells, providing new insights into PRRSV pathogenesis.

## Results

### RNA-seq and identification of differentially expressed lncRNAs

**Figure 1:**
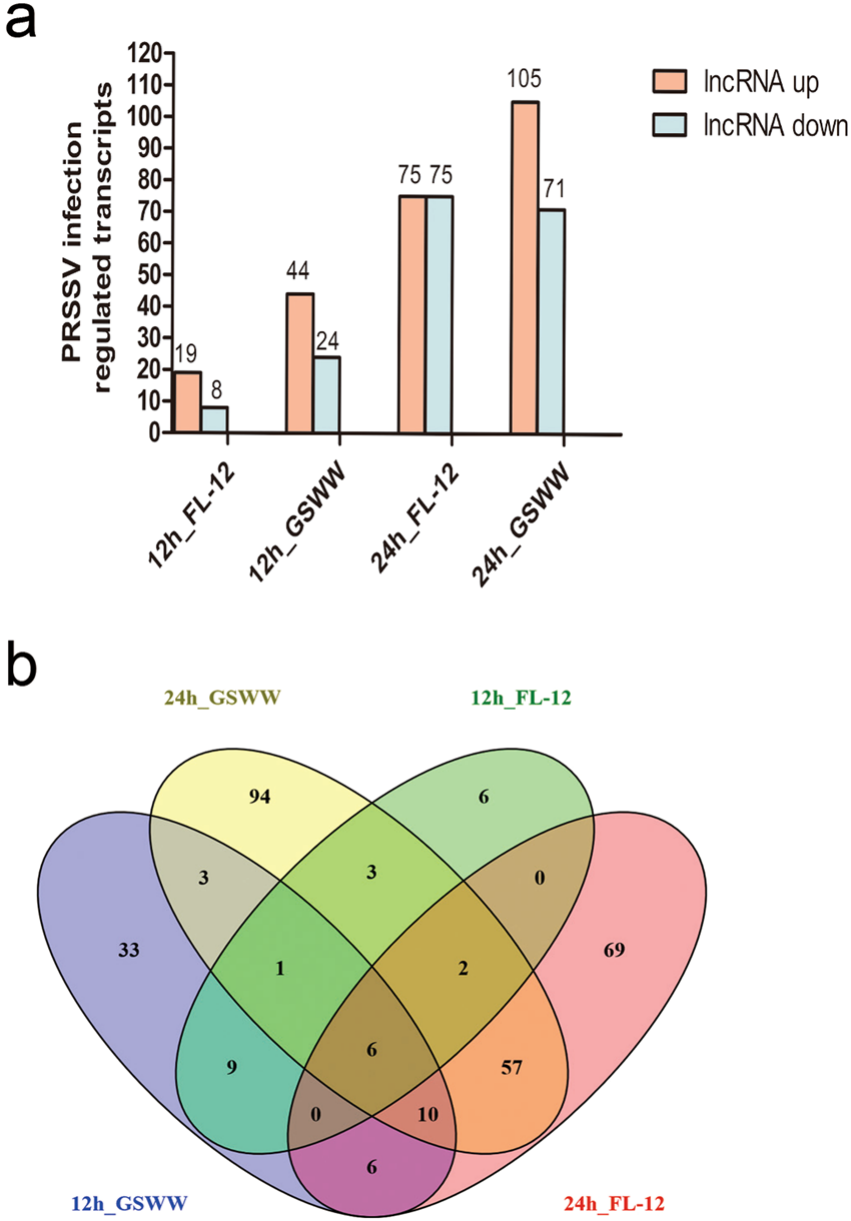
Numbers of differentially regulated genes after PRRSV infection. (**a**) Numbers of up- and down-regulated lncRNAs in four comparison groups (12h_GSWW vs. 12h_Mock, 12h_FL-12 vs. 12h_Mock, 24h_GSWW vs. 24h_Mock and 24h_FL-12 vs. 24h_Mock). The expression levels of lncRNAs were calculated by Cuffdiff on the basis of FPKM values. Differentially expressed lncRNAs were filtered by the cutoff p value ≤ 0.05. The lncRNAs showing the same alterations in two biological repeats were considered significantly differentially expressed. (**b**) Venn diagram illustrating common differentially expressed genes in four comparison groups.

We sequenced total RNA without rRNA for 12 samples including mock and GSWW- and FL-12 strain-infected PAMs at 12 and 24 hpi with duplicates (12h_MOCKA, 12h_GSWWA, 12h_FL-12A, 24h_MOCKA, 24h_GSWWA, 24h_FL-12A, 12h_MOCKB, 12h_GSWWB, 12h_FL-12B, 24h_MOCKB, 24h_GSWWB and 24h_FL-12B). After removing low-quality reads and adapter sequences, approximately 54 million clean reads were obtained for each sample. Clean reads were mapped to the pig reference genome (Sscrofa10.2) using TopHat and were assembled using Cufflinks. The protein coding potential of the novel transcripts was assessed using the Coding Potential Calculator (CPC). We identified 12,867 novel lncRNAs, 5545 were expressed in all the sequenced samples. The assembled sequences of the predicted lncRNAs are provided in Supplementary Table S1. In total, 299 differentially expressed lncRNAs from mock and PRRSV-infected cells were obtained by filtering the pairwise comparison results from Cuffdiff with p values ≤ 0.05 and removing those showed inconsistent alterations in duplicate groups. As shown in Fig. 1a, at 24 hpi, the GSWW infected group induced alterations in the expression levels of 176 lncRNAs, which was the greatest number among the four groups. We found that at both time points, GSWW infection induced more alteration of lncRNAs than FL-12. Sixteen and 76 lncRNAs were induced by both strains at 12 and 24 hpi, respectively (Fig. 1b and Supplementary Fig. S1). Differentially expressed lncRNAs are listed in Supplementary Table S2. We also analysed viral transcripts to find potential lncRNAs generated by PRRSV. However, we did not identify any lncRNAs produced by PRRSV.

### PRRSV infection altered lncRNA expression profiles in PAMs

**Figure 2:**
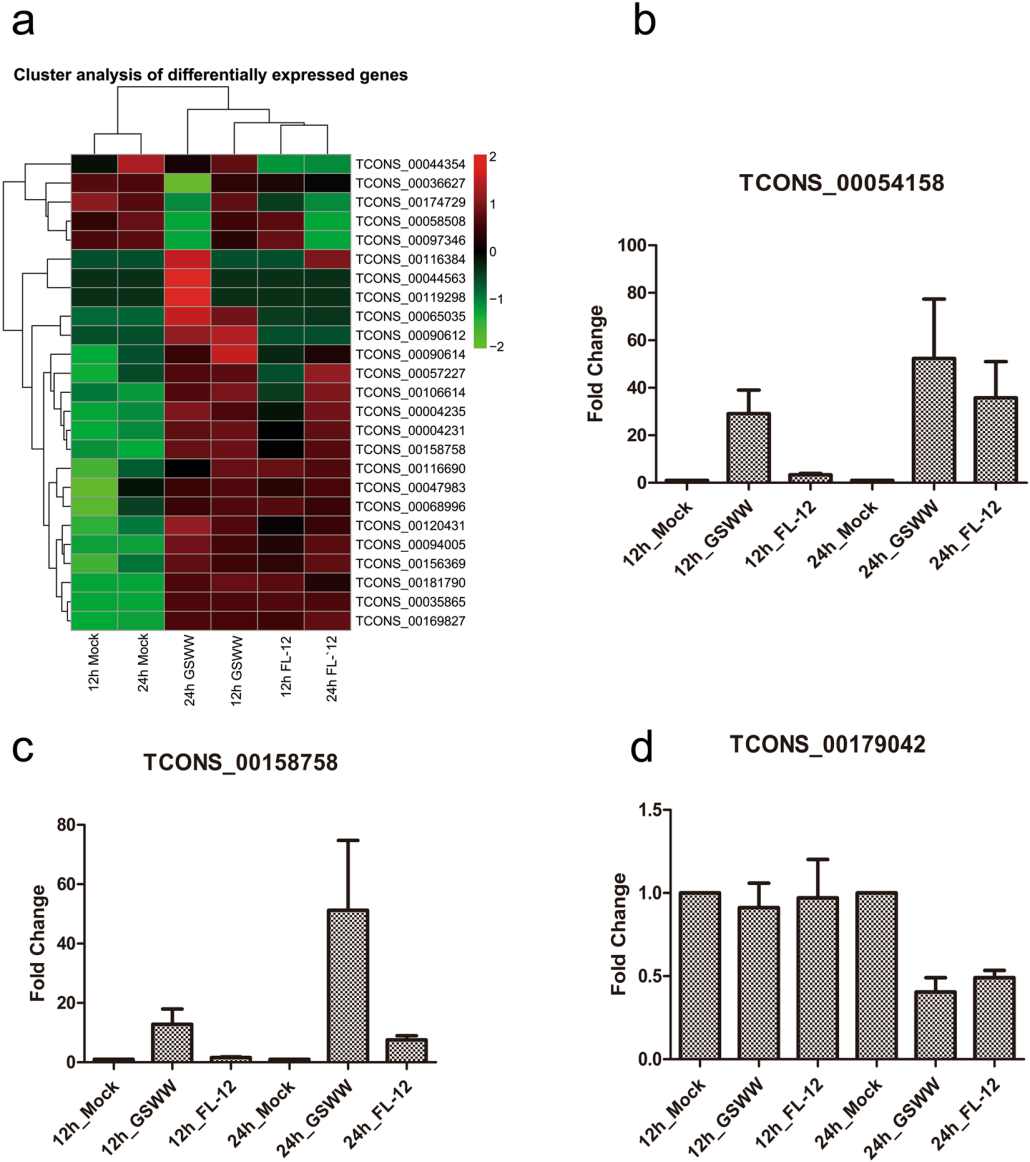
The expression levels of predicted lncRNAs. (**a**) Hierarchical heat map displaying transformed expression values (log2FPKM) for 25 significant differentially expressed lncRNAs. Red shows up-regulation and green shows down-regulation. (**b–d**) RT-qPCR results of differentially expressed lncRNAs after PRRSV infection by GSWW FL-12 at 12 and 24 hpi. Total RNA was extracted, and the first strand cDNA was synthesized using reverse transcriptase kit. Bar represents the mean of three independent experiments. Expression levels were normalized to GAPDH.

Figure 2a shows a heat map of 25 significantly affected lncRNAs in duplicate after PRRSV infection. The data represent hierarchical clustering of log2 fragments per kilobase of transcript per million mapped reads (FPKM) at each condition. We found that expression levels of several lncRNAs were elevated by infection of both strains as early as 12 hpi and remained highly expressed at 24 hpi, including TCONS_00035865, TCONS_00169827, and TCONS_00158758. The expression levels of TCONS_00090612, TCONS_00044563 and TCONS_00119298 were only increased by GSWW infection. Fewer down-regulated lncRNAs than up-regulated lncRNAs were identified in our RNA-seq data; this might be attributable to the low basal expression levels of lncRNAs. To validate the RNA-seq data, ten differentially expressed lncRNAs were selected for RT-qPCR analysis. The selected lncRNAs were differentially expressed in at least one treatment condition (Fig. 2 and Supplementary Fig. S2). The RT-qPCR results revealed that the expression patterns of most of the selected lncRNAs (9 of 10) were in strong agreement with the RNA-Seq data (Table 1). The FPKM values of all the differentially expressed lncRNAs are provided in Supplementary Table S3. We also detected PRRSV copy numbers by RT-qPCR, both strains replicated as early as 12 hours post-infection on PAM cells (Supplementary Fig. S3). As shown in Supplementary Fig. S3, at the two time points we detected, the copy numbers of GSWW and FL-12 were higher in biological repeat A than B, which may due to individual differences of the piglets.

**Table 1:** Correlation analysis of RNA-seq and RT-qPCR results.

Transcript ID		12 h Mock	12 h FL-12	12 h GSWW	24 h Mock	24 h FL-12	24 h GSWW	Correlation score
TCONS_00068991	FPKM	0.17	0.00	1.61	0.00	0.54	1.23	0.738
	qPCR	1.00	2.35	9.07	1.00	1.29	1.80	
TCONS_00090612	FPKM	0.00	0.00	4.22	0.00	0.00	1.73	0.937
	qPCR	1.00	1.48	9.26	1.00	1.56	1.75	
TCONS_00135148	FPKM	0.52	0.9	1.63	0.33	0.79	1.42	0.989
	qPCR	1.00	3.61	7.91	1.00	2.89	6.08	
TCONS_00054157	FPKM	2.18	12.26	22.83	2.3	60.83	35.28	0.851
	qPCR	1.00	3.02	31.90	1.00	68.58	88.63	
TCONS_00179042	FPKM	0.99	0.39	0.82	1.12	0.2	0.00	0.855
	qPCR	1.00	0.97	0.91	1.00	0.49	0.40	
TCONS_00054158	FPKM	3.24	11.42	32.5	3.47	85.03	44.8	0.807
	qPCR	1.00	3.36	29.1	1.00	35.82	52.41	
TCONS_00158758	FPKM	0.63	1.13	1.54	0.52	1.39	1.72	0.721
	qPCR	1.00	1.61	12.85	1.00	7.55	51.24	
TCONS_00052446	FPKM	0.00	0.00	0.99	0.00	0.00	0.00	0.998
	qPCR	1.00	1.31	15.68	1.00	2.83	1.56	
TCONS_00115146	FPKM	2.55	4.25	5.66	0.68	2.84	4.00	0.849
	qPCR	1.00	1.45	1.99	1.00	1.02	1.19	
TCONS_00106680	FPKM	0.00	0.00	0.00	4.45	0.00	0.00	0.399
	qPCR	1.00	0.68	0.65	1.00	1.02	1	

Pearson correlation coefficients were calculated by GraphPad Prism 5.0 software.

### Genomic features of lncRNAs in PAMs

**Figure 3:**
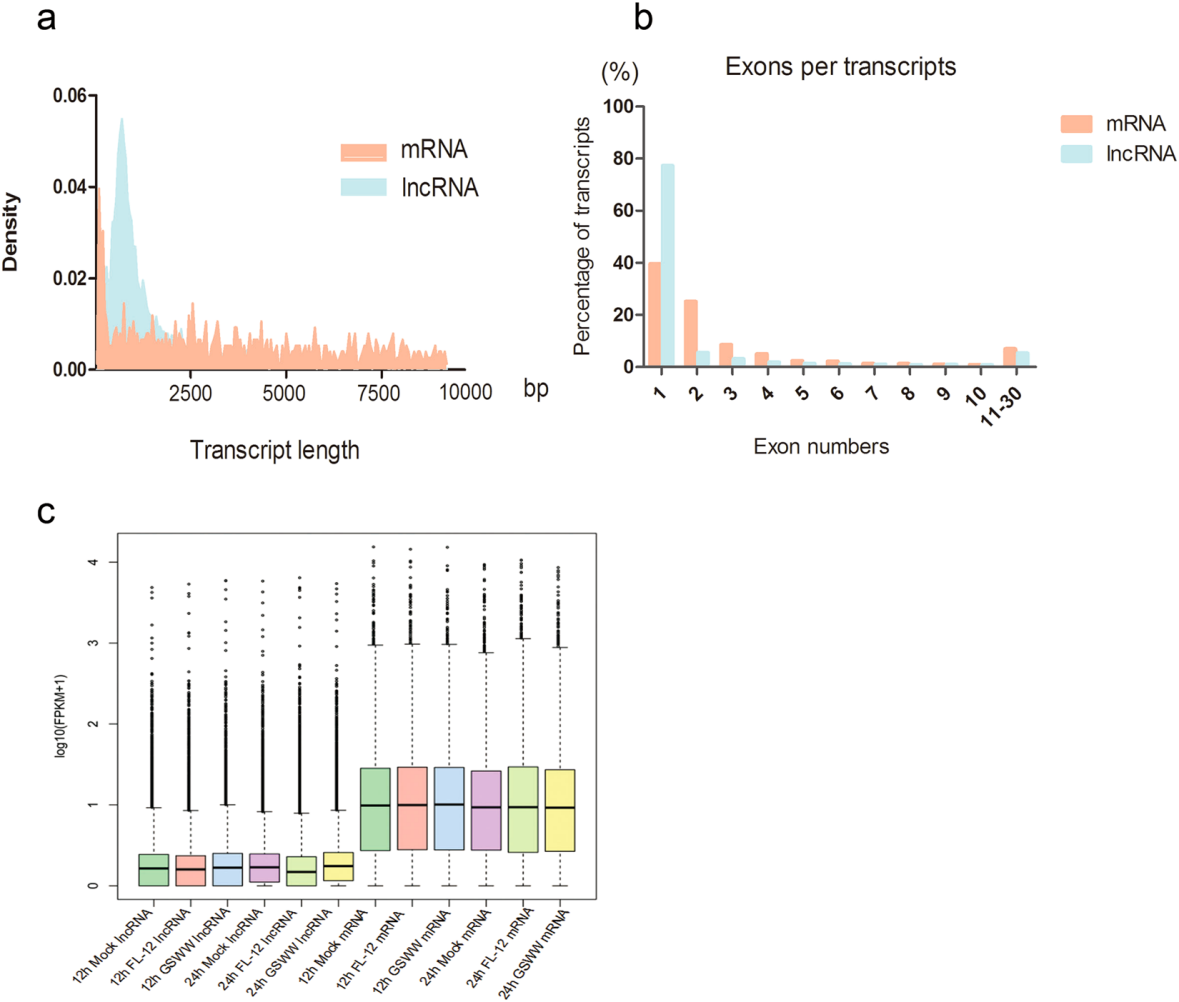
Genomic features of mRNAs and predicted lncRNAs. (**a**) Density map of length distribution of mRNAs and novel lncRNAs identified in RNA-Seq. (**b**) Exon number distributions of mRNAs and predicted lncRNAs in RNA-sequencing. (**c**) Boxplots demonstrating (showing minimum, 25%, 50%, 75% and maximum) the expression features of mRNAs and lncRNAs in each sample.

**Figure 4:**
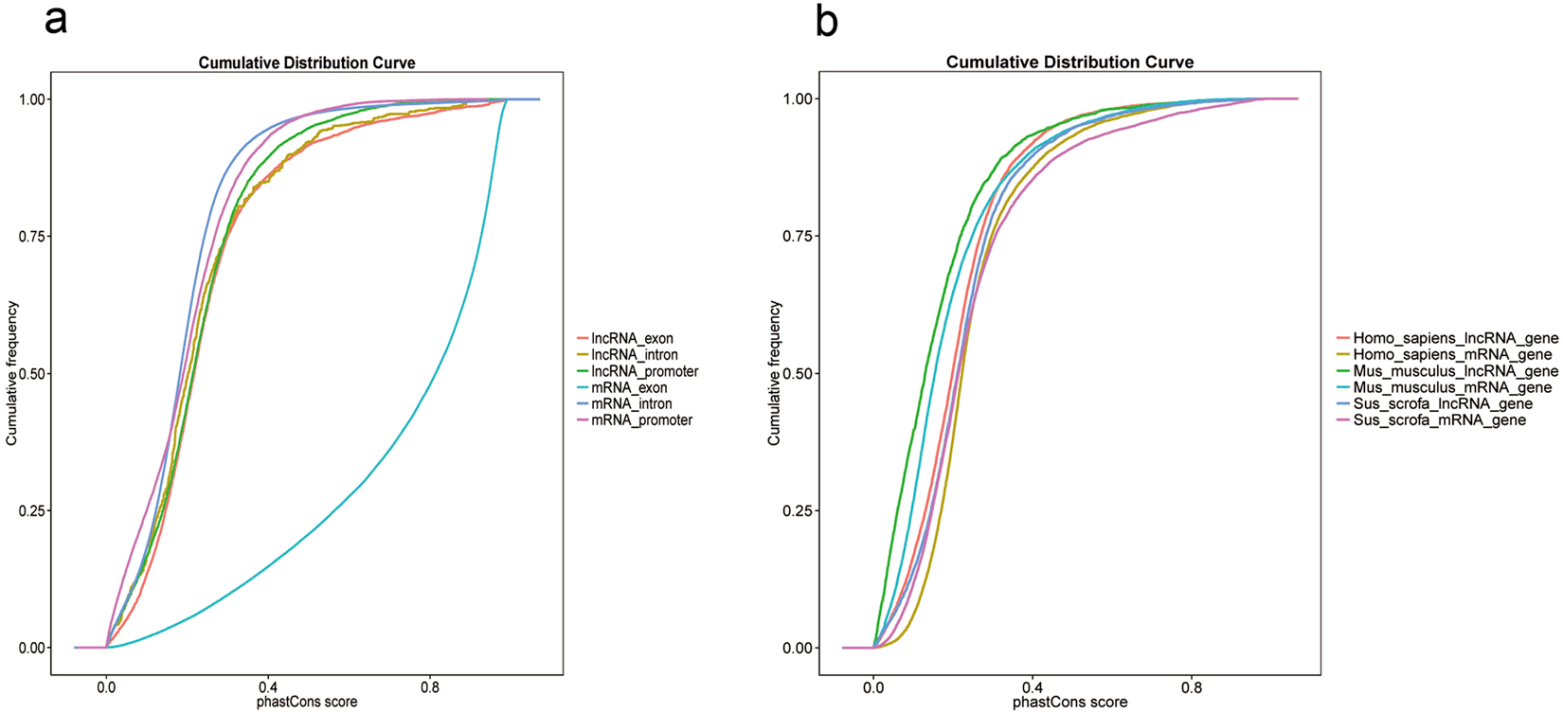
Conservation analysis of lncRNAs and mRNAs. PhyloFit was used to compute phylogenetic models for conserved and non-conserved regions among species. The model and HMM transition parameters were then input into phyloP to compute a set of conservation scores for lncRNA and coding genes. (**a**) Conservation scores of different regions of porcine lncRNAs and mRNAs identified in RNA-seq. (**b**) Conservation score of human, mouse, and porcine lncRNAs and mRNAs.

Although there is no specific sequence or structural feature to define lncRNAs, it has been reported that some genomic characteristics are shared by lncRNAs in vertebrates^30, 31^; they are generally shorter than protein-coding RNAs and have fewer exons (typically only one to three). We analysed the lncRNAs identified from our RNA-seq data for the genomic features mentioned above and found that, compared to protein-coding RNAs, lncRNAs are shorter in transcript length. The majority of lncRNAs were shorter than 2500 bp, while the distribution of mRNA transcript length was more even (Fig. 3a). Approximately 77.4% of the lncRNAs identified in our research had only one exon, and 39.6% of the annotated porcine mRNAs had one exon (Fig. 3b). We analysed the average expression levels of lncRNAs and mRNAs; boxplots illustrate that the expression levels of lncRNAs were lower than those of mRNAs (Fig. 3c), consistent with previous reports^32, 33^. We calculated the conservation scores of exons, introns, and promoters of mRNAs and lncRNAs, which were identified by RNA-seq using phastCon software. Exons of mRNAs were most conserved among these groups; in contrast to their protein-coding counterparts, the exons of lncRNAs were far less conserved (Fig. 4a). By comparing the conservation scores of human, mouse and pig lncRNAs and mRNAs, we found that in each species, lncRNAs were less conserved than mRNAs and that pig lncRNAs were more conserved than human and mouse lncRNAs (Fig. 4b).

### Functional predictions of lncRNAs

**Figure 5:**
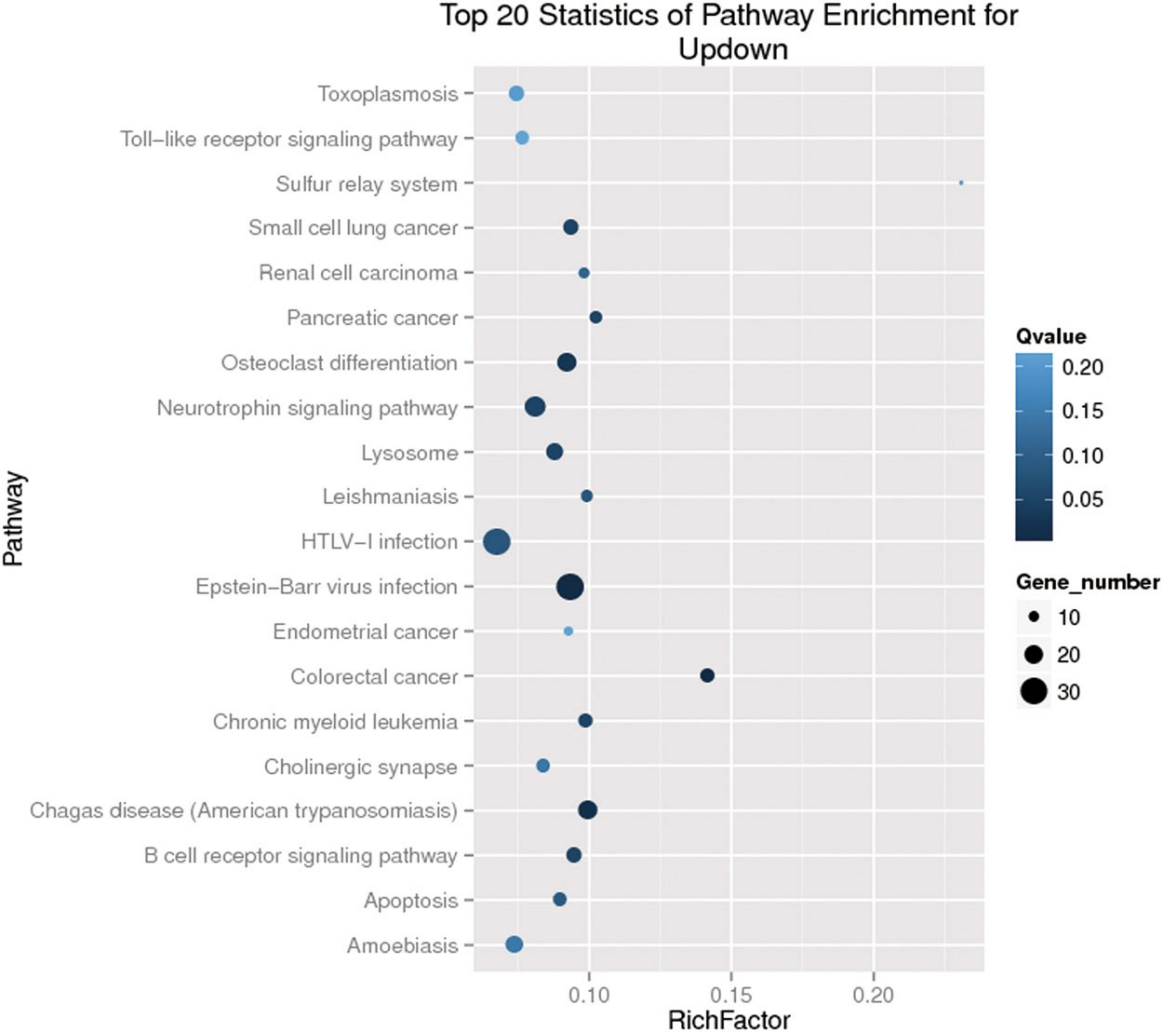
Scatter plot of KEGG pathway enrichment statistics. Top 20 statistics of pathways, enrichment in the KEGG database for neighbouring genes of predicted lncRNAs. Ten pathways had q values < 0.05 and were considered as significantly enriched pathways.

Although significant efforts have been made to understand the functions of lncRNAs, the roles of lncRNAs in the porcine immune system and their associated mechanisms remain mysterious. Unlike microRNAs or proteins, it is difficult to predict the functions of lncRNAs from their sequences or structures. According to some well-characterized models^34–36^, we categorized lncRNAs into four types to predict their functions: up- or downstream of mRNA genes, antisense transcripts of mRNAs, pre-miRNA, and the lncRNA family. It has been reported that lncRNAs can regulate transcription of their adjacent genes; some antisense lncRNAs interfere with gene expression in trans by modifying chromatin modification states. Using RNAplex, we found that 296 lncRNAs (Supplementary Table S4) could be annotated as antisense transcripts of mRNAs and that 1620 (Supplementary Table S5) were located adjacent to protein coding genes (2k). We performed Gene Ontology (GO) and Kyoto Encyclopaedia of Genes and Genomes (KEGG) enrichment analyses of these neighbour and antisense mRNAs. KEGG analysis demonstrated that most of the significantly (q value ≤ 0.05) enriched pathways in the neighbouring genes of lncRNAs belong to the “Epstein-Barr virus infection” pathway (Fig. 5). Many of the neighbouring genes correspond to compartments of the immune system, such as TLR2, TRAF6, IKK, IκBα, CD86, and TCR (Supplementary Table S6). We identified one lncRNA TCONS_00056284, as a potential pre-miRNA of miR-155 (Supplementary Table S7), which promotes type I IFN signalling in the antiviral innate immune response^37^. These results suggested that PRRSV infection might regulate the host immune response by inducing lncRNAs that act as regulatory elements in the porcine immune system. However, further efforts are required to confirm the present findings.

### Specificity of predicted lncRNA expression

**Figure 6:**
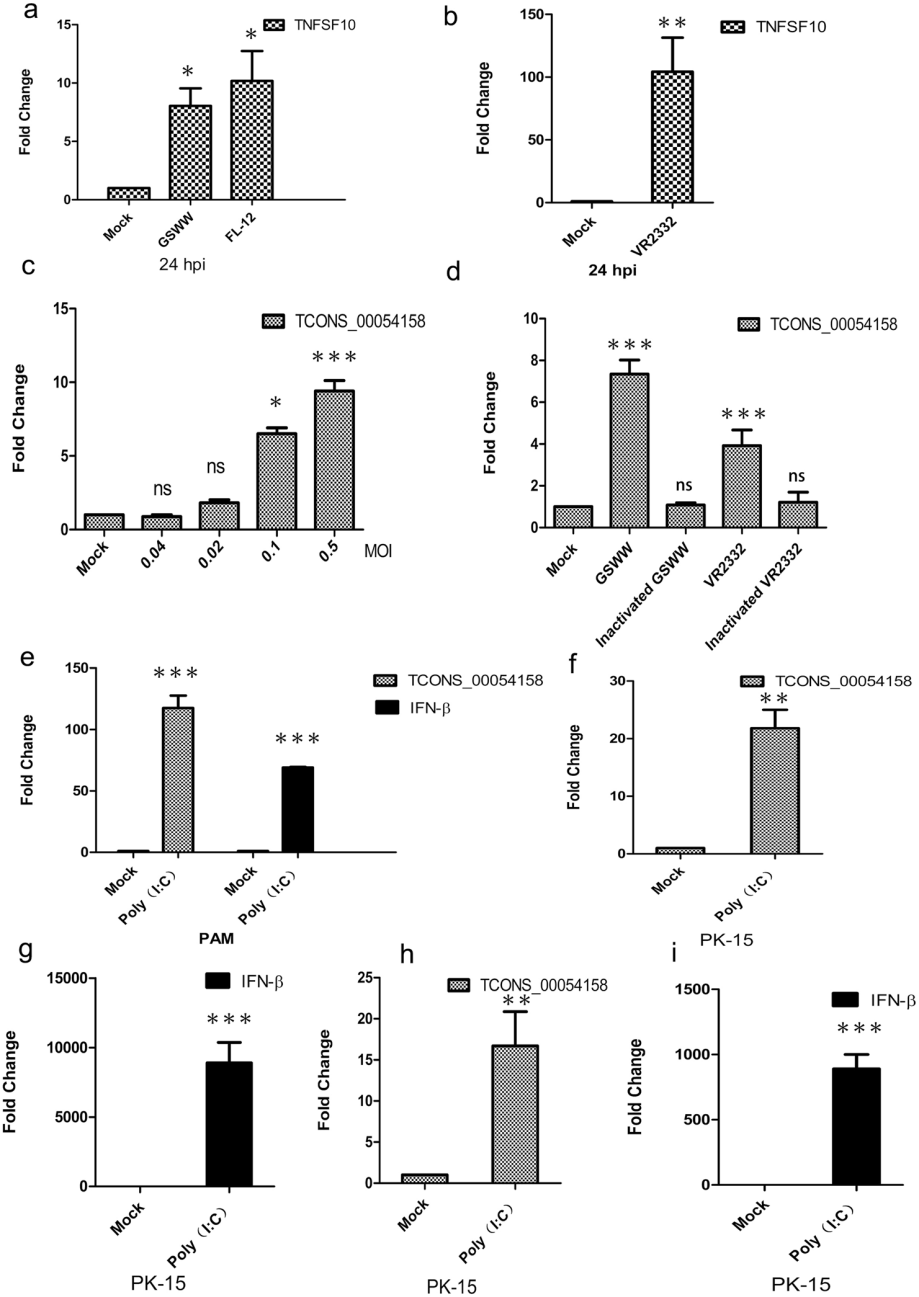
The expression levels of TNFSF10, TCONS_00158758 and IFN-β. Expression levels were normalized to GAPDH. Data are the means ± S.D. of three independent experiments. ***p < 0.001, **p < 0.01, *p < 0.05, one-way analysis of variance followed by Dunnett’s multiple comparison test or Student’s t-test was performed. (**a**) and (**b**). PAM cells were mock infected or infected with GSWW, FL-12 and VR2332 (at the MOI of 1). Total RNA was extracted 24 hpi and the first-strand cDNA was synthesized. RT-qPCR experiments were performed to detect TNFSF10 expression. (**c**) RT-qPCR results showing fold change in lncRNA TCONS_00158758 expression level in PAM cells at 24 hpi with gradient GSWW infection. A total of 10^7^ cells were plated in 25 cm^2^ flasks and mock infected or infected with 0.5 MOI, 0.1 MOI, 0.02 MOI, 0.004 MOI of GSWW per flask. (**d**) RT-qPCR data of lncRNA expression in PAM cells that were mock infected or infected with live GSWW, and VR2332 (at the MOI of 0.1) or heat-inactivated (56 °C, 120 min) viruses at 24 hpi. (**e**) PAM cells were treated with 100 µg/ml poly (I:C) for 24 hours. Total RNA was extracted and the first-strand cDNA was synthesized. RT-qPCR experiments were performed to detect the expression of IFN-β and lncRNA TCONS_00054158. (**f**) and (**g**) PK-15 cells were transfected with 2 µg/ml poly (I:C) for 24 hours. Total RNA was extracted and the first strand cDNA was synthesized. RT-qPCR experiments were performed to detect the expression of IFN-β and lncRNA TCONS_00054158. (**h**) and (**i**). PK-15 cells were treated with 100 µg/ml poly (I:C) for 24 hours. Total RNA was extracted and the first strand cDNA was synthesized. RT-qPCR experiments were performed to detect the expression of IFN-β and lncRNA TCONS_00054158.

As shown in Fig. 1b, six lncRNAs were differentially expressed upon infection of both strains at the two time points. Five were up-regulated, namely TCONS_00047044, TCONS_00054157, TCONS_00054158, TCONS_00158758 and TCONS_0169827; and TCONS_00179042 was down-regulated. We found that the genomic location of TCONS_00054158 was adjacent (40 K) to the gene for TNFSF10 (tumour necrosis factor (ligand) superfamily, member 10, also known as TRAIL). TNFSF10 is a cytokine that induces apoptosis, previous reports have shown that it is up-regulated after PRRSV infection^27^. Our RT-qPCR results showed that infection of GSWW, FL-12 or VR2332 could induce TNFSF10 expression (Fig. 6a and b). Therefore, lncRNA TCONS_00054158 may play a regulatory role in the expression of TNFSF10. To confirm that the altered expression of lncRNA TCONS_00054158 was caused by viral infection, we infected PAM cells with various titers of the GSWW strain and detected TCONS_00054158 expression levels. The RT-qPCR data demonstrated that TCONS_00054158 expression increased in line with viral titer, verifying the specificity of virally induced expression of TCONS_00054158 (Fig. 6c). We also determined whether various virulent strains could induce lncRNA expression and found that the infection of the less virulent vaccine strain VR2332 also increased TCONS_00054158 expression in PAM cells (Fig. 6d). Furthermore, we found that the expression of TCONS_00054158 could not be induced by inactivated PRRSV (Fig. 6d). Treatment with poly (I:C), the synthetic analogue of viral double-stranded RNA, induced elevation of TCONS_00054158 and IFN-β expression levels in PAM cells (Fig. 6e). In PK-15 cells, both transfection and treatment with poly (I:C) induced expression of TCONS_00054158 and IFN-β (Fig. 6f–i).

## Discussion

In 2007, HP-PRRSV strains emerged in China and Southeast Asia; the infected pigs were characterized by high fever, high morbidity, and high mortality and resulted in tremendous economic losses. The PRRSV pathogenesis has been intensively studied over the past decade. Although remarkable achievements have been made, the underlying mechanism remains elusive. Several groups have investigated host-pathogen interactions at the transcriptome and proteome levels. These studies revealed that PRRSV infection induced expression of host immune response genes such as Toll-like receptors, NF-κB, and IRFs and pro-inflammatory cytokines such as IL-1β and IL-8. The microRNAomes of PRRSV-infected pig lungs and PAMs were detected using deep sequencing^38–40^ and revealed that miR-147 and ssc**-**miR-30d-R_1 play regulatory roles in PRRSV infection and the antiviral responses. Numerous studies have demonstrated the essential roles of lncRNAs in the modulation of viral infection. NRAV regulates antiviral responses through suppression of the initial transcription of IFN-stimulated genes^9^. LncRNA-CMPK2 was up-regulated in hepatitis C virus (HCV)-infected human liver and negatively regulated the IFN response^41^. However, the roles of lncRNAs in the regulation of viral-host interactions during PRRSV infection remain unclear. To the best of our knowledge, this is the first report that focuses on the expression profiles of lncRNAs in PAM cells after PRRSV infection.

Suppression of the host immune system remains one of the most challenging obstacles in PRRS vaccine development^42^. Insights into the immune suppression mechanism of PRRSV will help in developing novel anti-virus therapeutic strategies. In this study, we predicted hundreds of novel lncRNAs whose expression levels were altered after PRRSV infection in piglet macrophages. The highly pathogenic strain GSWW induced more differentially expressed lncRNAs than the typical North American strain FL-12, indicating that lncRNAs might contribute to the pathogenesis of the highly virulent strain. The functions of these lncRNAs remain mysterious. Despite “transcriptional noise”, some lncRNAs may take part in the regulation of viral-host interactions. The KEGG enrichment analysis of the neighbouring genes of lncRNAs indicated that some predicted targets are essential regulators of the anti-viral immune response. The lncRNAs may regulate neighbouring gene transcription by recruiting chromatin-regulatory complexes. Conservation analysis revealed that lncRNAs in PAM cells are less conserved than mRNAs. This result correlates with previous findings in other vertebrates and in porcine endometrium^43^ and skeletal muscle^44^. Most lncRNAs exert their function through their secondary structure rather than primary sequence; therefore the structure but not the sequence may be under selection pressure, which may explain the lack of conservation of lncRNAs among species.

The expression profiles of lncRNAs are cell-type-, tissue-, development stage-, and disease-specific, which may indicate their potential as biomarkers in disease diagnosis. Many lncRNAs have been correlated with human diseases such as cancer and neurological diseases. Since lncRNA could positively or negatively regulate gene transcription, it serves as a potential therapeutic target. There is emerging commercial interest in the potential of lncRNAs as drug targets. The high evolution rate of the PRRSV sequence and the lack of cross-protection of genetically various strains is the main challenge in PRRSV vaccine development. In this study, we found that the predicted lncRNA TCONS_00054158 was induced in PAM cells by a highly pathogenic field-isolated strain from China, a typical North American strain and a modified live vaccine strain. However, heat- inactivated virus could not induce TCONS_00054158 expression. In addition, poly (I:C) treatment significantly induced TCONS_00054158 expression in PAM and PK15 cells, indicating that up-regulation of TCONS_00054158 expression might be a common feature of RNA virus infection in pig. TNF-α signalling is mainly activated in macrophages and plays important roles in inflammation. It has been reported that HP-PRRSV infection impaired LPS and poly (I:C) induced TNF-α release^45^. Furthermore, PRRSV infection inhibits apoptosis in early stages and accelerates apoptosis in the late stages of infection, which may be a strategy for the virus to generate as many virions as possible^1^. TNFSF10 (TRAIL) induces apoptosis in a caspase-dependent manner. Hepatitis B virus-triggered autophagy can target TNFRSF10B for degradation to limit the TNFSF10 response^46^. It is possible that TCONS_00054158 modulates apoptosis during PRRSV infection by regulating TNFSF10 expression. Loss- and gain-of function studies are needed to explore the biological significance of this lncRNA expression. Understanding the roles of lncRNAs in PRRSV infection and pathogenesis might provide novel diagnosis and therapeutic strategies.

## Material and Methods

### Sample preparation

Two 4-week-old healthy pigs with similar genetic backgrounds were purchased from the same commercial herd. Pigs were euthanized, and lungs were separated and washed with phosphate-buffered saline (PBS). PAMs were harvested by centrifuging and frozen in foetal bovine serum (FBS) with 10% dimethyl- sulfoxide (DMSO) in liquid nitrogen. Two different pathogenic PRRSV stocks, GSWW15 (HP-PRRSV) and FL-12 (the rescued viruses from full-length cDNA clone) were used in this study. Viruses were propagated on Marc-145 cells and filtered and stored at −70 °C. Twenty-four hours before infection, 1 × 10^7^ PAM cells were plated in 25 cm^2^ flasks and maintained in RMPI-1640 medium supplemented with 10% FBS, 100 IU penicillin, and 100 µg streptomycin/ml. PAM cells were infected with GSWW and FL-12 at a multiplicity of infection (MOI) of 1 for 12 or 24 hours; control groups were mock-infected with Marc-145 culture medium without PRRSV infection. The cell culture medium was discarded, cells were washed with PBS once, and cell pellets were collected using cell scrapers and harvested by centrifuging. Cell pellets were immediately frozen in liquid nitrogen and stored at −70 °C until total RNA was extracted. Animal experiments were performed according to the rules described by the Animal Ethics Procedures and Guidelines of the People’s Republic of China and were approved by the Animal Ethics Committee of LVRI, Chinese Academy of Agricultural Sciences (permission number: SYXK-GAN-2010-0001).

### Total RNA extraction

Total RNA was extracted using RNeasy Kit following the manufacturer’s protocol (Qiagen). The quantity and purity of total RNA were evaluated by Nanodrop 2000, and the integrity of the RNA was analysed by the Bioanalyser 2100 system (Agilent Technologies, CA, USA).

### Preparation of the RNA-seq library

A total amount of 3 μg RNA was utilized in the RNA sample preparations. First, ribosomal RNA was removed with an Epicentre Ribo-zero™ rRNA Removal Kit (Epicentre, Madison,WI, USA), and residual RNAs were cleaned using ethanol precipitation. Twelve sequencing libraries were generated using the rRNA-depleted RNA with a NEBNext® Ultra™ Directional RNA Library Prep Kit for Illumina® (NEB, USA). The quality of the libraries was evaluated on an Agilent Bioanalyser 2100 system.

### Data analysis

Raw data were processed using the SOAPnuke software (http://soap.genomics.org.cn/) developed by BGI to filter adaptor sequences and remove the low-quality reads to obtain clean, high-quality data. To completely remove the remaining rRNA reads, the clean reads were mapped to an rRNA reference using the short-read alignment software SOAPaligner/SOAP2^47^ before further bioinformatics analysis have proceeded. Then the reads were mapped to the porcine reference genome (Sscrofa10.2), GSWW15 and FL-12 genomes respectively using TopHat (2.0.10). The mapped reads for each sample were independently assembled using Cufflinks (2.1.1).

### Prediction of novel lncRNAs

The assembled transcripts were filtered by FPKM >0.5, coverage >1 and length >200 bp to remove background noise. To detect novel lncRNAs, the filtered transcripts were compared to the reference annotation by utilizing Cuffcompare^48^. Five candidate categories of transcripts that may contain novel transcripts were extracted, including “A transfrag falling entirely within a reference intron”, “Potentially novel isoform”, “Generic exonic overlap with a reference transcript”, “Unknown, intergenic transcript”, and “Exonic overlap with reference on the opposite strand”^49^. After each sample was assembled, Cuffmerge was utilized to merge all assemblies together, which helps to reconstruct genes that may not fully be recovered due to insufficient sequencing depth in each replicate. At this step, novel identified isoforms were also integrated with known isoforms into complete gene models^50^. Novel lncRNAs were predicted using the CPC^51^, a Support Vector Machine-based classifier used to assess the protein-coding potential of a transcript based on six biologically meaningful sequence features. The first three features assess the extent and quality of the ORF in a transcript. Three other features were derived from parsing the output of a BLASTX search (using the transcript as query, *E*-value cutoff 1*e*-10) against the UniProt Reference Clusters (UniRef90), which were developed as a nonredundant protein database with a 90% sequence identity threshold.

### Differentially expressed gene analyses

The expression level of each transcript was calculated with Cuffdiff, which uses the Cufflinks transcript quantification engine to calculate gene and transcript expression on the basis of FPKM values. Differentially expressed transcripts were filtered by the cutoff p value ≤ 0.05. The transcripts showed the same regulation patterns in pair-wise cooperation groups in two biological repeats that were considered significantly differentially expressed.

### Annotation and function prediction

RNAplex^36^ is a software program that rapidly searches for short interactions between two long RNAs and was used to reveal the potential antisense lncRNA-mRNA interactions. The program predicts the best base-base pairing using a minimum free energy algorithm according to its thermodynamic features. LncRNAs, which are classified as located in an “unknown region” in Cuffcompare were annotated as up-or downstream of a gene. These lncRNAs likely regulate transcription of neighbouring genes. The lncRNAs were aligned to miRBase^52^ to detect potential pre-miRNA, and hit coverage above 90% were selected. miRPara^53^ were used to predict probable miRNA. The predicted novel lncRNAs were categorized into different non-coding RNA families using INFERNAL, which classifies noncoding RNAs, their conserved primary sequence and RNA secondary structure using multiple alignments (MSAs), consensus secondary structure annotation and covariance models (CMs)^54^.

### GO and KEGG analyses

GO enrichment analyses were performed to identify specific pathways or functions significantly enriched in antisense mRNAs and neighbour genes of predicted lncRNAs. KEGG was used to perform pathway enrichment analysis of antisense and adjacent genes for the predicted lncRNAs. Q values < 0.05 were considered as significantly enriched pathways.

### Conservation analyses

The phast software package (v1.3) was used for phylogenetic analysis. PhastCons is a conservation scoring and identifying program for conserved elements. Then phyloFit was utilized to compute phylogenetic models for conserved and non-conserved regions among species; the model and HMM transition parameters were set for phastCons to compute the conservation scores of the lncRNAs and mRNAs^43, 55^.

### Poly (I:C) treatment and transfection and inactivated virus preparation

PAM cells and PK-15 cells were cultured in 6-well plates for 24 hours before treatment, 100 µg/ml Poly (I:C) (Sigma) or sterilized ddH_2_O was added, total RNA was extracted 24 hours after treatment. PK-15 cells were plated in 6-well plates for 24 hours before transfection, 2 µg/ml Poly (I:C) was transfected using effectene reagent (Qiagen). Total RNA was extracted 24 hours after transfection. Viruses was heated at 56 °C for 2 hours to be inactivated^56^.

### Real-time RT-PCR and statistical analysis

Total RNA from PAM cells or PK-15 cells was extracted by RNeasy mini kit (Qiagen). First-strand cDNA was synthesized using a reverse transcriptase Kit (TaKaRa). Real-time PCR was performed on an Applied Biosystems 7500 Real-Time PCR System. SYBR® Green PCR Master Mix (TaKaRa) was used; the cycling conditions included a single initial cycle (95 °C for 30 s), followed by 40 cycles (95 °C for 5 s, 60 °C for 30 s, and 72 °C for 30 s). The specificity of the amplified products was analysed using dissociation curves and Sanger sequencing. Relative expression levels of lncRNAs were normalized to GAPDH using the 2^−ΔΔCt^ method. For detection of PRRSV copy numbers, Real Time One Step RT-PCR was performed. The cycling condition included two step for reverse transcription (42 °C for 5 min, 95 °C for 10 s), followed by 40 cycles (95 °C for 5 s, 60 °C for 34 s). The results represent the averages from three independent experiments. Real-time primers and TaqMan probe sequences are shown in Supplementary Table S8. Data from three independent experiments were analysed using Student’s t-test or one-way ANOVA followed by Dunnett’s multiple comparison test (compare all groups to the control group). All data are demonstrated as the means ± S.D. (*p < 0.05, **p < 0.01, ***p < 0.001).

## Electronic supplementary material

Supplementary information

Supplementary Table S1

Supplementary Table S2

Supplementary Table S3

Supplementary Table S4

Supplementary Table S5

Supplementary Table S6

Supplementary Table S7
