# Supplementary material for: Genome-wide analysis of long noncoding RNA profiling in PRRSV-infected PAM cells by RNA sequencing
Source: Sci Rep. 2017 Jul 10;7:4952. doi: 10.1038/s41598-017-05279-z (PMC5504012; doi:10.1038/s41598-017-05279-z)
Supplement: Supplementary file 1 — Supplementary information [file 41598_2017_5279_MOESM1_ESM.pdf]

Supplementary information

Genome-wide analysis of long noncoding RNA profiling in PRRSV-infected PAM cells by RNA sequencing

Jing Zhang, Pu Sun, Lipeng Gan,Weijie Bai, Zhijia Wang, Dong Li, Yimei Cao, Yuanfang Fu, Pinghua Li, Xingwen Bai, Xueqing Ma, Huifang Bao, Yingli Chen, Zaixin Liu*, Zengjun Lu*

State Key Laboratory of Veterinary Etiological Biology, OIE/National Foot-and-Mouth Disease Reference Laboratory of China, Lanzhou Veterinary Research Institute, Chinese Academy of Agricultural Sciences, Lanzhou 730046, China

*Corresponding author.

Tel: +86-931-8343390; Fax: +86-931-8340977.

E-mail: liuzaixincaas@163.com; luzengjun@caas.cn

Corresponding address: Lanzhou Veterinary Research Institute, Chinese Academy of Agricultural Sciences, Xujiaping No.1, Yanchangpu, Lanzhou, Gansu 730046, China

Figure S1

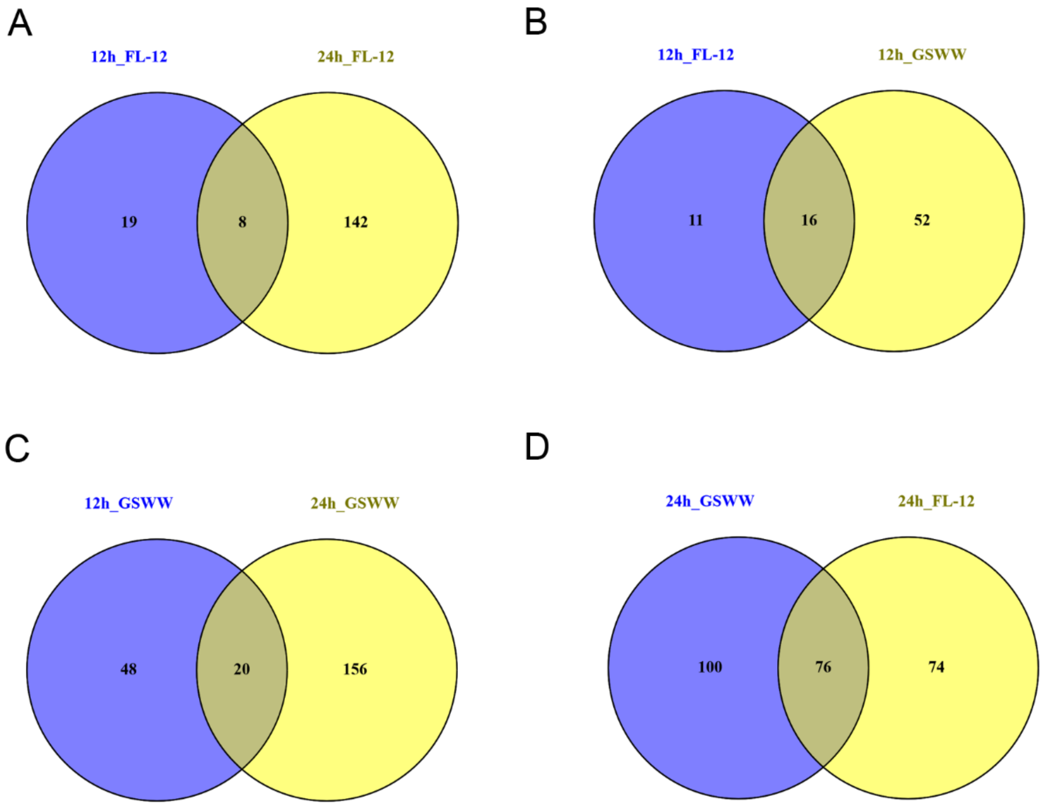

Figure S1. Venn diagram showing common differentially expressed genes in four comparison groups.

Figure S2

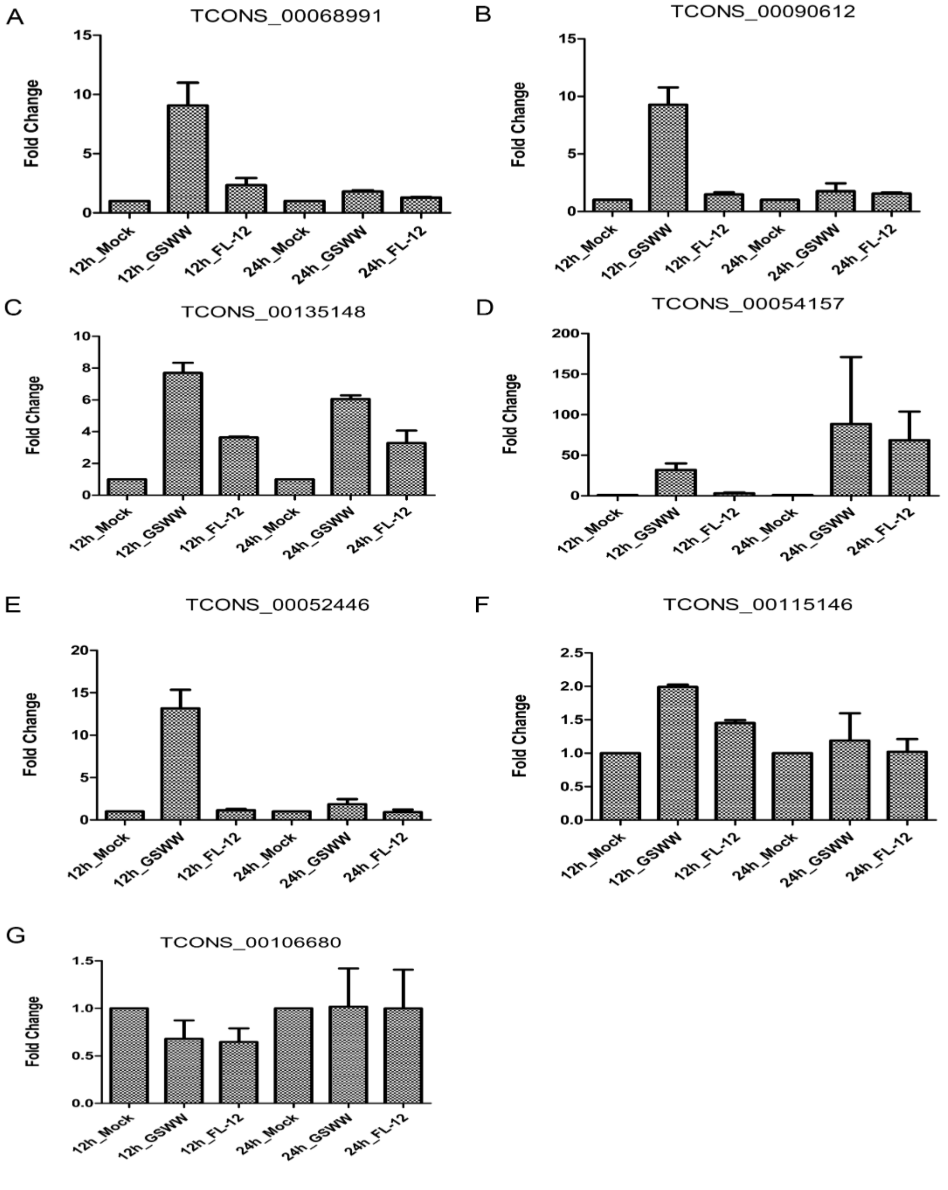

Figure S2. RT-qPCR validation of lncRNA expression. RT-qPCR results of differentially expressed lncRNAs after PRRSV infection by GSWW FL-12 at 12 hpi and 24 hpi. Total RNA was extracted, and the first strand cDNA was synthesized using reverse transcriptase Kit. Bar represents the mean of three independent experiments. Expression levels were normalized to GAPDH.

Figure S3

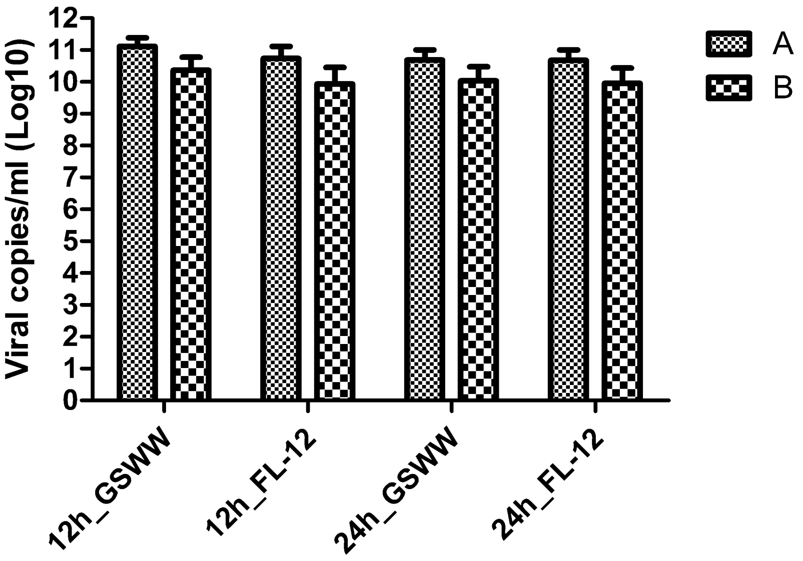

Figure S3. Viral copies/ml of PRRSV were detected by RT-qPCR. The expression levels of ORF6 of PRRSV were detected by Real Time One Step RT-PCR Kit (TAKARA). A total of 100 ng RNA/sample was used for each reaction. The quantity of PRRSV copies numbers was determined by measuring 10-fold serial dilutions of the PRRSV RNA standards ranging from 1×10^3 to 1×10^8 copies/µl in triplicates.

Supplementary Table S1 presents the assembled sequences of the predicted lncRNAs.

Supplementary Table S2 lists differentially expressed lncRNAs.

Supplementary Table S3 shows lncRNAs annotated as antisense transcripts of mRNAs.

Supplementary Table S4 lists lncRNAs located adjacent to protein coding genes.

Supplementary Table S5 provides neighbouring genes of lncRNAs enriched in KEGG pathways.

Supplementary Table S6 shows that lncRNA TCONS_00056284 was predicted to be a potential pre-miRNA of miR-155.

Supplementary Table S7 lists real-time primers and TaqMan probe sequences used in this study.
